# Impaired neurocognitive and psychomotor performance in patients with inflammatory bowel disease

**DOI:** 10.1038/s41598-019-50192-2

**Published:** 2019-09-24

**Authors:** Ivana Tadin Hadjina, Piero Marin Zivkovic, Andrija Matetic, Doris Rusic, Marino Vilovic, Diana Bajo, Zeljko Puljiz, Ante Tonkic, Josko Bozic

**Affiliations:** 1 Department of Gastroenterology, University Hospital of Split, Split, Croatia; 2 Department of Pathophysiology, University of Split School of Medicine, Split, Croatia; 3 Department of Pharmacy, University of Split School of Medicine, Split, Croatia; 4 Department of Rheumatology and Clinical Immunology, University Hospital of Split, Split, Croatia; 5 Department of Internal Medicine, University of Split School of Medicine, Split, Croatia

**Keywords:** Neurological manifestations, Inflammatory bowel disease

## Abstract

Limited evidence exists regarding cognitive and psychomotor function in patients with inflammatory bowel disease (IBD). Therefore, we aimed to compare the neurocognitive and psychomotor function of 60 IBD patients with 60 age/sex-matched controls. Computer-based instrument Complex Reactinometer Drenovac (CRD) was used for assessment of cognitive domains: convergent thinking (simple mathematical tasks; CRD-11), perceptive abilities (light signal position discrimination; CRD-311) and sophisticated operative thinking (complex psychomotor coordination; CRD-411). The most important analyzed parameters were total test solving time (T_TTS_); minimal time of particular test solving (T_MIN_) and total number of wrong reactions (N_ER_). Performance in all three cognitive tests showed statistically significantly longer T_TTS_ and T_MIN_ in IBD patients (*P* < 0.05), while there was no significant difference in N_ER_. Aforementioned findings were adjusted for BMI, age and duration of education. Our study has shown impaired neurocognitive and psychomotor function in IBD patients compared to controls, especially in mental processing speed and mental endurance of perceptive abilities, convergent thinking and complex operative thinking.

## Introduction

Inflammatory bowel disease (IBD) is an idiopathic chronic inflammatory disorder of the gastrointestinal system with serious acute and chronic complications^1^. The rising prevalence of Crohn’s disease (CD) and ulcerative colitis (UC) is the highest in the most developed countries. However, the epidemiological shift and accelerating incidence of IBD in developing countries is concerning, and as such represent an enormous burden in global health^2,3^.

The pathogenesis of IBD involves numerous etiologic factors and complex pathophysiologic mechanisms associated with all organ systems. Likewise, the close interrelationship of IBD and the nervous system has been proposed in a multitude of studies^4,5^. Specifically, the negative effects of neuropsychological disorders on gastrointestinal functioning have been clinically observed for a long time^4^. Still, the impact of the nervous system on the pathophysiology of IBD is one of the least studied factors^5^.

It is reasonable to expect bidirectional effects on neurological function in patients with IBD. Previous studies have proposed that patients with IBD exhibit harmful neuropsychological repercussions, although the exact pathophysiological mechanisms are not fully clarified^4–6^. The importance of gut-brain-microbiome interaction has recently been emphasized, providing new insights into IBD pathophysiology and therapeutic options^5^. Moreover, it is well-known that IBD represents a general proinflammatory state with major cytokine disbalance, which has numerous neurological consequences^1^. Likewise, other factors including an impaired hypothalamic-pituitary-adrenal (HPA) axis could interfere with neurophysiologic function^7,8^. It has been shown that CD patients exhibit a cognitive deficit in the Stroop test which was proposed to be the consequence of a dysfunctional HPA axis^9^. Besides, patients with IBD are often malnourished, which can possibly impair cognitive and psychomotor function^10,11^. Similarly, concurrent mood disorders, long-term medication use, chronic fatigue and chronic pain syndrome can alter the cognitive profile in the IBD population^12^.

One of the most important neurophysiological outcomes is cognitive and psychomotor function^5^. Several studies have questioned cognitive performance in the IBD population, yet the results are inconsistent^6,13–19^. Nonetheless, scarcely available evidence suggests deteriorations in specific domains and sub-domains of cognitive function, which could consequently interfere with the daily functioning of IBD patients^13,15–17^.

Consequently, several studies have shown that IBD patients often suffer from psychosocial and economic difficulties – impaired interpersonal relationships, higher risk for unemployment, job limitations and work abseenteism^20^. Additionally, numerous studies have shown adverse effects of IBD on health-related quality of life (HRQoL), which is highly affected by daily cognitive and psychomotor abilites^21^. In fact, high-grade evidence studies have found that psychological and cognitive behavioural therapy can positively influence HRQoL in patients with IBD^22^. Finally, cognitive impairment increases the risk of all-cause mortality^23^.

Therefore, it is of clinical importance to establish whether IBD patients suffer from cognitive and psychomotor consequences. As mentioned, studies evaluating cognitive and psychomotor function in patients with IBD are insufficient and show inconsistent results^6,13–19^. Furthermore, co-existence of multiple comorbidities disturbs the establishment of an independent relationship^18^. Moreover, methodological differences interfere with direct result comparison and valid conclusions. Available questionnaire-based studies implicate psychometric bias, while research utilizing more complex neuropsychological tests vary at the sample level and are inconclusive^6,13–19^. Nevertheless, whether IBD *per se* alters cognitive and psychomotor function is still unknown. Therefore, further studies on computer-generated neuropsychological tests, comprising both CD and UC patients, are needed.

Hence, the aim of this study was to determine the psychomotor and cognitive function of the patients with IBD in comparison to healthy subjects determined by computerized neuropsychological tests CRD-series. To our knowledge, a CRD-series test was not yet utilized for the evaluation of cognitive and psychomotor function in the IBD population.

## Methods

### Ethical considerations

All subjects were informed about the goal, procedures, and course of this study. Before the onset, the study protocol was approved by the Ethics Committees of the University of Split School of Medicine and the University Hospital of Split. All participants provided written informed consent and all procedures were carried out in accordance with the 1964 Declaration of Helsinki and its later amendments, as well as the Good Clinical Practice guidelines from the International Conference on Harmonisation.

### Study design

This cross-sectional study was conducted in the Department of Pathophysiology at the University of Split School of Medicine and in the Department of Gastroenterology at the University Hospital of Split from December 2017 to July 2018.

### Subjects

This cross-sectional study included 60 patients with IBD evaluated in the Department of Gastroenterology at the University Hospital of Split as well as 60 age- and sex-matched healthy control subjects recruited from the general population. The diagnosis of IBD was established according to the recent guidelines by the European Crohn’s and Colitis Organisation and the European Society of Gastrointestinal and Abdominal Radiology^24^. Inclusion criteria were: disease duration of at least one year; stable disease activity in the previous 3 months; and age 18–65 years. Exclusion criteria were: diagnosis of depression, anxiety, chronic fatigue or other mood disorder; evident signs and symptoms of depression, anxiety or chronic fatigue; positive history of cognitive or psychomotor impairment; hypothyroidism; sleep disorder; neurological disorder; neuromuscular disease; therapy with blood transfusions, corticosteroid or other medications with known effects on neuropsychological functions during 3 months prior to the study onset; use of psychoactive medications; and alcohol consumption of more than 40 grams/day and substance abuse. Control subjects were screened for the presence of abdominal pain, defecation-related symptoms, changes in the frequency and form of stool, according to the Rome IV criteria for inflammatory bowel syndrome, as well as any other abdominal symptom suggestive of lactose and gluten intolerance. Overall, we have prescreened 70 patients and 65 control subjects, out of which 10 patients and 5 control subjects were excluded from the study due to inadequate criteria fulfillment and lack of free time.

### Clinical assessment

Relevant clinical data were extracted from patients’ medical records. Other clinical information were gathered using a specific questionnaire. All subjects included in the study denied the presence of depressive symptoms in a self-reported questionnaire.

### Assessment of disease activity

The simple endoscopic score for Crohn’s disease (SES-CD) is a specifically designed tool for endoscopic evaluation of CD activity. Empirical cut-off values for result interpretation vary between studies, but we have used thresholds according to the majority of studies: inactive disease ≤2; mild activity 2–7; moderate activity 7–16; and severe disease ≥16^25^.

The Crohn’s disease activity index (CDAI) is a well-established mathematic instrument for the assessment of CD activity based on several patient-reported symptoms in a 7-day period, clinician-reported signs and laboratory results. Threshold scores for interpretation are: clinical remission <150; mild to moderate activity 150–450; and severe disease >450^26^.

The Harvey-Bradshaw index (HBI), a simplified version of the CDAI, was developed to alleviate a clinician’s assessment of CD activity. Empirical cut-off values are mostly set to: clinical remission <5; mild activity 5–7; moderate activity 8–16; and severe disease >16^26^.

The ulcerative colitis endoscopic index of severity (UCEIS) is a quantitative indicator of mucosal inflammation based on the specific colonoscopic findings. According to the final score, disease stage is classified as remission (0–1), mild (2–4), moderate (5–6), or severe (7–8). It provides satisfactory intraobserver and interobserver reliability for the assessment of UC severity^27^.

The Mayo endoscopic score (MES) is a reliable instrument for UC staging based only on endoscopic evaluation. Disease can be classified in several categories: 0 – inactive disease (normal mucosa); 1 – mild disease (erythema, decreased vascular pattern, and mild friability); 2 – moderate disease (marked erythema, absence of vascular patterns, friability and erosions); 3 – severe disease (spontaneous bleeding and ulceration)^28^.

The Mayo score/disease activity index (Mayo/DAI) is a clinical score used for the assessment of UC activity. The summary score provides good correlation with UC severity: <2 remission (with no individual subscore >1); 3–5 mild; 6–10 moderate; and 10–12 severe^29^.

### Assessment of cognitive and psychomotor function

The Complex Reactionmeter Drenovac (CRD) is a well-established psychodiagnostic instrument, specifically designed for the detailed evaluation of cognitive and psychomotor function. The concept of CRD-series tests is based on chronometric principles^30–34^. We have utilized 3 representative tests in our study, namely CRD-311 (light signal position discrimination), CRD-11 (simple mathematical tasks) and CRD-411 (complex psychomotor coordination tasks). The aforementioned tests enable a focused assessment of convergent thinking, perceptive abilities and sophisticated operative thinking, respectively^30^.

The CRD-311 test contains 60 single tasks in which subjects must provide an accurate answer by pressing a switch below the corresponding randomly illuminating light-emitting diode. The CRD-11 consists of mathematical symbols (addition or subtraction), randomly illuminating light-emitting diodes and corresponding numbers positioned on the test dashboard, which are used for the construction of simple mathematical tasks, and requires test solving by pressing the button that corresponds to the correct result of the constructed mathematical operations. Finally, the CRD-411 test requires simultaneous limb coordination (arm and leg) in reaction to the random illumination of two light-emitting diodes, each corresponding to one limb^30^.

The most important parameters derived from the CRD-series test include basic time variables (total test solving time [T_TTS_]; minimal time of particular test solving [T_MIN_]; average time of particular test solving [T_AVER_]; time of reactions in the first test phase [T_1_]; time of reactions in the second test phase [T_2_]; time of reactions in the third test phase [T_3_]; time of reactions in the fourth test phase [T_4_]), as well as response stability and variability (total ballast time [TB]) and error variables (total number of wrong reactions [N_ER_]). The aforementioned parameters are descriptors of mental processing speed and mental endurance (T_TTS_; T_AVER_; T_1_; T_2_; T_3_; T_4_), maximal cognitive potential (T_MIN_), accuracy of mental processing and alertness (N_ER_), as well as “wasted time”/stability/fluctuation (TB) of cognitive function^30,31^. Higher test scores (T_TTS_; T_MIN_) indicate poorer cognitive and motor performance^31^.

We focused on testing specific cognitive domains that were previously tested in the IBD population as well in other chronic diseases^30–34^. Before the test initiation, all subjects received detailed instructions and were able to perform a trial test. The test was conducted during the morning in a sufficiently illuminated, translucent and quiet room, to eliminate possible distractions. The test sequence was conducted according to the test complexity, from the most simple to the most complicated. Testing was conducted by an experienced technician who was “blinded” to the group type (patients or control subjects) during the experiment. Importantly, all subjects were instructed to sleep at least 8 hours during the night prior to the testing. Similarly, smoking and alcohol consumption was forbidden on the testing day for all subjects.

### Sample collection and laboratory analysis

Venous blood samples were taken after the performance of the CRD-series test through a polyethylene catheter inserted into a forearm vein. Hemoglobin levels (Hb) and high sensitivity C-reactive protein (hs-CRP) were analyzed with routine laboratory methods in the same biochemical laboratory and by the same specialist in medical biochemistry. Faecal calprotectin (FC) concentrations had been measured from stool samples after appropriate collection, following standard laboratory protocols, within one week after psychometric testing.

### Statistical analysis

MedCalc statistical software (Ostend, Belgium; version 11.5.1.0) for Windows was used for statistical data analysis. We assessed the normality of data distribution graphically and by the Kolmogorov-Smirnov test. Data were expressed as means ± standard deviation (SD) for continuous parametric variables and as whole numbers and percentages for categorical variables. The Student’s t-test was used for the analysis of independent continuous data. We used the Chi-square test for the comparison of sex differences, smoking status, alcohol and coffee consumption between the IBD and control group. Moreover, we compared differences in disease activity, extraintestinal manifestations and surgical history between CD and UC subgroups by the Chi-square test. Multiple linear regression analysis with a stepwise selection algorithm was used to determine the relative importance of independent variables (Hb, hs-CRP, FC, BMI, age, duration of education, CDAI, Mayo/DAI) in the prediction of CRD parameters. Variables that did not reach statistical significance were excluded from the model. Analysis of covariance (ANCOVA) was used to compare group differences in CRD parameters controlling for important covariates including age, duration of education and body mass index (BMI). The aforementioned variables were included in the ANCOVA model due to their relevance to cognitive function. Levene’s test for the equality of variances was performed prior to the ANCOVA analysis. All assumptions for the ANCOVA analysis were confirmed. Finally, to investigate the difference in test solving times within and between groups according to the different test phases, a two-factor (group; test phase) repeated measures ANOVA was carried out. We estimated sphericity using the Greenhouse-Geisser method. If the estimate of sphericity (epsilon) wass greater than 0.75, the assumption of sphericity was met. If not, we used the Greenhouse-Geisser correction factor. The statistical significance was defined as *P* < 0.05.

## Results

### Basic anthropometric and related characteristics

There were no differences in the age, gender, anthropometric characteristics, smoking status, and coffee and alcohol consumption between our study groups (*P* > 0.05). However, the laboratory analysis revealed lower Hb (139.17 ± 17.29 vs. 146.83 ± 15.16, *P* = 0.012) and higher hs-CRP levels (13.19 ± 35.65 vs. 1.20 ± 1.23, *P* = 0.012) in IBD patients. Finally, control subjects did not differ according to their education level (P = 0.396) (Table 1).

**Table 1:** Comparison of basic anthropometric and related characteristics between the IBD and control group.

Parameter		IBD group (n = 60)	Control group (n = 60)	*P*
Age (years)		40.37 ± 14.90	39.12 ± 12.17	0.616^a^
Sex	Male	36 (60.00%)	41 (68.33%)	0.446^b^
	Female	24 (40.00%)	19 (31.67%)	
Body weight (kg)		76.72 ± 14.17	81.86 ± 17.38	0.079^a^
Body height (cm)		177.08 ± 0.09	179.64 ± 0.10	0.146^a^
BMI (kg/m^2^)		24.37 ± 3.68	25.15 ± 3.67	0.066^a^
Active smoking		11 (18.33%)	13 (21.67%)	0.892^b^
hs-CRP		13.19 ± 35.65	1.20 ± 1.23	0.012^a^
Hb		139.17 ± 17.29	146.83 ± 15.16	0.012^a^
Alcohol consumption		23 (38.33%)	32 (53.00%)	0.099^b^
Coffee consumption		25 (41.67%)	33 (55.00%)	0.094^b^
Duration of education (years)		14.44 ± 1.81	14.75 ± 2.16	0.396^a^

Continuous data are presented as mean ± standard deviation and categorical data are presented as number (percentage).

BMI – body mass index; hs-CRP – high sensitivity C-reactive protein; Hb – hemoglobin.

^a^Student t-test for independent samples.

^b^Chi squared test.

### Disease and laboratory characteristics of the IBD group

Most of the observed patients suffered from CD (58.30%, N = 35). According to the international clinical scores, both subgroups of patients were in clinical remission from the mild form of disease (Crohn’s disease: CDAI – remission; HBI – remission; Ulcerative colitis: Mayo/DAI – mild activity), while endoscopic scoring systems displayed disease of moderate activity (Crohn’s disease: SES-CD – moderate activity; Ulcerative colitis: UCEIS – moderate activity; MES – moderate activity). Patients with CD had more extraintestinal manifestations and surgical treatments (62.86%, N = 22 vs. 24.00%, N = 6, *P = *0.003 and 34.28%, N = 12 vs. 4.00%, N = 1, *P* = 0.047, respectively), while there was no difference in disease duration, disease activity and laboratory parameters (Table 2).

**Table 2:** Comparison of disease characteristics between different IBD categories.

Parameter	Crohn’s disease (n = 35, 58.30%)	Ulcerative colitis (n = 25, 41.70%)	*P*
SES-CD	11.09 ± 8.90	—	—
CDAI	63.51 ± 62.44	—	—
HBI	3.10 ± 2.22	—	—
UCEIS	—	6.05 ± 2.04	—
MES	—	2.42 ± 0.84	—
Mayo/DAI	—	4.67 ± 2.94	—
Disease duration (years)	10.08 ± 6.21	8.04 ± 9.15	0.339^a^
Self-reported perception of disease activity	21 (60.00%)	16 (64.00%)	0.789^b^
Extraintestinal manifestations	22 (62.86%)	6 (24.00%)	0.003^b^
Positive history of surgical treatment	12 (34.28%)	1 (4.00%)	0.047^b^
hs-CRP	8.78 ± 24.62	13.65 ± 40.25	0.517^a^
Hb	138.67 ± 18.10	136.91 ± 20.37	0.686^a^
FC	475.63 ± 807.29	565.76 ± 959.07	0.650^a^

Continuous data are presented as mean ± standard deviation and categorical data are presented as number (percentage).

SES-CD – simple endoscopic score for Crohn’s disease; CDAI – Crohn’s disease activity index; HBI – Harvey-Bradshaw index; UCEIS – ulcerative colitis endoscopic index of severity; MES – Mayo endoscopic score; Mayo/DAI – Mayo score/disease activity index for ulcerative colitis; hs-CRP – high sensitivity C-reactive protein; Hb – hemoglobin; FC – faecal calprotectin.

^a^Student t-test for independent samples.

^b^Chi squared test.

### Outcomes of cognitive performance

#### Test of perceptive abilities (CRD-311 – light signal position discrimination)

The CRD-311 essentially offers insight into perceptive abilities, comprising several components – detection accuracy, perception speed, spatiovisual orientation and simple psychomotoric reaction. The analysis of CRD-311 test results showed significantly longer T_TTS_ (36.13 ± 6.43 vs. 33.71 ± 7.17, P = 0.029), T_MIN_ (0.44 ± 0.08 vs. 0.41 ± 0.10, P = 0.020), T_3_ (7.10 ± 1.27 vs. 6.61 ± 1.32, P = 0.019) and T_4_ (7.05 ± 1.26 vs. 6.55 ± 1.19, *P* = 0.012) in IBD patients, while there was no statistically significant difference in the other variables between observed groups. After adjustment for confounding variables, all variables remained statistically significant with the addition of T_AVER_, which appeared longer in IBD patients (0.60 ± 0.11 vs. 0.56 ± 0.12, *P* = 0.007) (Table 3).

**Table 3:** Comparison of CRD parameters between the IBD and control group.

Test type	Parameter	IBD group (n = 60)	Control group (n = 60)	*P* ^a^	*P* ^b^	Cohen’s *d*^c^
CRD 311	T_TTS_ (s)	36.13 ± 6.43	33.71 ± 7.17	0.029	0.008	−0.355
	T_MIN_ (s)	0.44 ± 0.08	0.41 ± 0.10	0.020	0.001	−0.331
	T_AVER_ (s)	0.60 ± 0.11	0.56 ± 0.12	0.056	0.007	−0.347
	T_1_ (s)	7.93 ± 1.64	7.33 ± 2.16	0.055	0.052	−0.313
	T_2_ (s)	7.14 ± 1.28	6.74 ± 1.71	0.098	0.089	−0.265
	T_3_ (s)	7.10 ± 1.27	6.61 ± 1.32	0.019	0.002	−0.378
	T_4_ (s)	7.05 ± 1.26	6.55 ± 1.19	0.012	0.001	−0.408
	N_ER_	0.01 ± 0.11	0.08 ± 0.49	0.225	0.435	0.197
	TB (s)	9.39 ± 2.87	9.11 ± 3.49	0.595	0.755	−0.088
CRD 11	T_TTS_ (s)	145.91 ± 57.37	124.14 ± 37.06	0.006	0.004	−0.451
	T_MIN_ (s)	2.28 ± 0.66	2.01 ± 0.56	0.007	0.004	−0.441
	T_AVER_ (s)	3.73 ± 1.25	3.28 ± 0.90	0.013	0.008	−0.413
	T_1_ (s)	29.83 ± 14.56	24.99 ± 9.16	0.015	0.020	−0.398
	T_2_ (s)	31.93 ± 12.48	26.12 ± 8.06	<0.001	<0.001	−0.553
	T_3_ (s)	28.55 ± 11.76	25.08 ± 9.08	0.043	0.029	−0.330
	T_4_ (s)	28.44 ± 14.84	24.32 ± 7.29	0.033	0.031	−0.352
	N_ER_	4.04 ± 5.74	2.69 ± 2.46	0.064	0.074	−0.306
	TB (s)	66.10 ± 41.91	53.88 ± 23.64	0.029	0.062	−0.359
CRD 411	T_TTS_ (s)	46.33 ± 19.15	39.65 ± 14.25	0.015	0.009	−0.396
	T_MIN_ (s)	0.57 ± 0.14	0.51 ± 0.12	0.011	0.005	−0.460
	T_AVER_ (s)	1.00 ± 0.31	0.91 ± 0.23	0.035	0.023	−0.330
	T_1_ (s)	8.61 ± 5.08	6.55 ± 2.06	0.001	0.001	−0.531
	T_2_ (s)	8.42 ± 5.03	6.99 ± 2.63	0.030	0.024	−0.356
	T_3_ (s)	8.45 ± 3.29	8.04 ± 3.90	0.475	0.621	−0.114
	T_4_ (s)	12.02 ± 5.50	10.12 ± 4.52	0.020	0.022	−0.377
	N_ER_	10.64 ± 8.34	8.25 ± 7.90	0.070	0.040	−0.294
	TB (s)	26.46 ± 16.24	21.67 ± 12.53	0.042	0.048	−0.330

Data are presented as mean ± standard deviation.

T_TTS_ – total test solving time; T_MIN_ – minimal time of particular test solving; T_AVER_ – average time of particular test solving; T_1_ – time of reactions in the first test phase; T_2_ – time of reactions in the second test phase; T_3_ – time of reactions in the third test phase; T_4_ – time of reactions in the fourth test phase; N_ER_ – total number of wrong reactions; TB – total ballast time; s – seconds.

^a^Student t-test for independent samples.

^b^ANCOVA model adjusted for BMI, age and duration of education.

^c^Cohen’s *d* for effect size.

#### Test of convergent thinking (CRD-11 – simple mathematical tasks)

The CRD-11 test consists of simple arithmetic operations and is a measure of decision making speed, problem solving ability and handling difficult situations. Compared to the control group, IBD patients achieved statistically significantly worse values in most parameters of the CRD-11 test, except for N_ER_ which did not reach statistical significance. Moreover, after controlling for covariates, all parameters retained statistical significance, with the exception of TB (Table 3).

#### Test of sophisticated operative thinking (CRD-411 – complex psychomotor coordination)

The CRD-411 measures speed of perception, complex mental processing, detection accuracy, spatiovisual orientation, concentration and complex psychomotoric reaction times. All results from the CRD-411 test panel were statistically significantly deteriorated in the observed IBD sample compared to control subjects, except for T3 and N_ER_ which lacked statistical significance. Importantly, after controlling for confounding variables, none of the parameters lost statistical significance. Additionally, N_ER_ proved to be higher in IBD patients (10.64 ± 8.34 vs. 8.25 ± 7.90, P = 0.040) (Table 3).

### Analysis of the IBD subgroups

There was no difference in CRD test parameters between patients with CD and UC, except in T_MIN_ of CRD-311 which was longer among patients with UC (0.47 ± 0.08 vs. 0.43 ± 0.08, *P* = 0.045) (Table 4).

**Table 4:** Comparison of CRD parameters between CD and UC patients.

Test type	Parameter	Crohn’s disease (n = 35)	Ulcerative colitis (n = 25)	*P* ^a^
CRD 311	T_TTS_ (s)	35.39 ± 6.17	37.13 ± 6.73	0.234
	T_MIN_ (s)	0.43 ± 0.08	0.47 ± 0.08	0.045
	T_AVER_ (s)	0.59 ± 0.10	0.62 ± 0.11	0.231
	T_1_ (s)	7.65 ± 1.32	8.30 ± 1.94	0.075
	T_2_ (s)	7.04 ± 1.26	7.28 ± 1.32	0.407
	T_3_ (s)	6.91 ± 1.19	7.35 ± 1.35	0.124
	T_4_ (s)	6.97 ± 1.28	7.16 ± 1.23	0.515
	TB (s)	9.59 ± 2.61	9.11 ± 3.22	0.465
CRD 11	T_TTS_ (s)	142.93 ± 43.93	149.93 ± 72.24	0.593
	T_MIN_ (s)	2.27 ± 0.57	2.29 ± 0.78	0.922
	T_AVER_ (s)	3.68 ± 1.11	3.78 ± 1.44	0.728
	T_1_ (s)	28.52 ± 11.66	31.61 ± 17.78	0.351
	T_2_ (s)	31.74 ± 11.23	32.18 ± 14.16	0.879
	T_3_ (s)	27.39 ± 8.23	30.11 ± 15.30	0.311
	T_4_ (s)	27.25 ± 8.63	30.05 ± 20.52	0.407
	TB (s)	63.34 ± 27.60	69.82 ± 56.05	0.498
CRD 411	T_TTS_ (s)	45.23 ± 19.66	47.81 ± 18.63	0.556
	T_MIN_ (s)	0.56 ± 0.12	0.58 ± 0.15	0.386
	T_AVER_ (s)	0.97 ± 0.25	1.05 ± 0.37	0.279
	T_1_ (s)	8.40 ± 3.92	8.90 ± 6.37	0.669
	T_2_ (s)	8.16 ± 5.50	8.76 ± 4.36	0.602
	T_3_ (s)	8.00 ± 3.00	9.06 ± 3.61	0.157
	T_4_ (s)	12.11 ± 6.20	11.91 ± 4.46	0.873
	TB (s)	25.76 ± 17.57	27.39 ± 14.44	0.660

Data are presented as mean ± standard deviation.

T_TTS_ – total test solving time; T_MIN_ – minimal time of particular test solving; T_AVER_ – average time of particular test solving; T_1_ – time of reactions in the first test phase; T_2_ – time of reactions in the second test phase; T_3_ – time of reactions in the third test phase; T_4_ – time of reactions in the fourth test phase; TB – total ballast time; s – seconds.

^a^Student t-test for independent samples.

### Multiple linear regression analysis of CRD parameters with hemoglobin, high sensitivity C-reactive protein and faecal calprotectin in the total IBD group

There were no significant effects of Hb, hs-CRP and FC in prediction of CRD parameters in the IBD patients (Table 5).

**Table 5:** Multiple linear regression analysis of selected variables as independent predictors of CRD parameters in the IBD patients.

Test type		Hb	hs-CRP	FC	Age	Duration of education	Overall
		B^a^ (t^b^) *P*					
CRD 311	T_TTS_	ns	ns	ns	360.86 (10.63) <0.001	−126.46 (−5.14) 0.028	R^2^ adjusted = 360.86
							F ratio = 113.00
							*P* < 0.001
	T_MIN_	ns	ns	ns	4.34 (9.11) <0.001	ns	R^2^ adjusted = 0.525
							F ratio = 82.90
							*P* < 0.001
	N_ER_	ns	ns	ns	ns	ns	R^2^ adjusted = n/a
							F ratio = n/a
							*P* = n/a
	TB	ns	ns	ns	100.70 (4.92) <0.001	ns	R^2^ adjusted = 0.239
							F ratio = 24.18
							*P* < 0.001
CRD 11	T_TTS_	ns	ns	ns	360.86 (10.63) <0.001	−14.51 (−2.14) 0.042	R^2^ adjusted = 0.602
							F ratio = 113.00
							*P* < 0.001
	T_MIN_	−7.64 (−2.20) 0.031	ns	ns	31.77 (7.39) <0.001	ns	R^2^ adjusted = 0.419
							F ratio = 27.66
							*P* < 0.001
	N_ER_	ns	ns	ns	ns	ns	R^2^ adjusted = n/a
							F ratio = n/a
							*P* = n/a
	TB	ns	ns	ns	1364.69 (4.36) <0.001	ns	R^2^ adjusted = 0.196
							F ratio = 19.00
							*P* < 0.001
CRD 411	T_TTS_	ns	ns	ns	979.84 (8.45) <0.001	ns	R^2^ adjusted = 0.488
							F ratio = 71.00
							*P* < 0.001
	T_MIN_	ns	ns	ns	5.68 (6.19) <0.001	ns	R^2^ adjusted = 0.335
							F ratio = 38.28
							*P* < 0.001
	N_ER_	ns	ns	ns	0.21 (3.17) 0.002	ns	R^2^ adjusted = 0.109
							F ratio = 10.07
							*P* = 0.002
	TB	ns	ns	ns	781.21 (7.54) <0.001	ns	R^2^ adjusted = 0.430
							F ratio = 57.00
							*P* < 0.001

T_TTS_ – total test solving time; T_MIN_ – minimal time of particular test solving; N_ER_ – total number of wrong reactions; TB – total ballast time; Hb – hemoglobin; hs-CRP – high sensitivity C-reactive protein; FC – faecal calprotectin.

^a^B-value indicates regression coefficient of the independent variable.

^b^t-value indicates t-statistic value.

### Multiple linear regression analysis of BMI, age, duration of education and disease activity indices as independent predictors of CRD parameters in the IBD group

The multiple linear regression model on CD patients showed that age was the strongest independent predictor of cognitive performance in all CRD-tests, while other variables generally did not exhibit significant correlation (Table 6). Similarly, age was confirmed as a statistically significant independent predictor for most CRD parameters, among UC patients (Table 7).

**Table 6:** Multiple linear regression analysis of selected variables as independent predictors of CRD parameters in the CD patients.

Test type		BMI	Age	Duration of education	CDAI	Overall
		B^a^ (t^b^) *P*				
CRD 311	T_TTS_	ns	254.94 (3.98) <0.001	−114.13 (−3.14) 0.037	ns	R^2^ adjusted = 0.304
						F ratio = 15.82
						*P* < 0.001
	T_MIN_	ns	3.56 (4.33) <0.001	ns	ns	R^2^ adjusted = 0.343
						F ratio = 18,79
						*P* < 0.001
	N_ER_	ns	ns	ns	ns	R^2^ adjusted = n/a
						F ratio = n/a
						*P* = n/a
	TB	ns	61.24 (1.96) 0.057	ns	ns	R^2^ adjusted = 0.077
						F ratio = 3.84
						*P* = 0.059
CRD 11	T_TTS_	-3866.97 (-2.40) 0.022	2075.68 (4.03) <0.001	ns	ns	R^2^ adjusted = 0.296
						F ratio = 8.16
						*P* = 0.001
	T_MIN_	ns	26.36 (5.86) <0.001	ns	ns	R^2^ adjusted = 0.575
						F ratio = 16.36
						*P* < 0.001
	N_ER_	ns	ns	ns	ns	R^2^ adjusted = n/a
						F ratio = n/a
						*P* = n/a
	TB	ns	337.43 (2.06) 0.047	−18.79 (−3.67) 0.076	ns	R^2^ adjusted = 0.087
						F ratio = 4.23
						*P* = 0.049
CRD 411	T_TTS_	ns	647.43 (3.31) 0.002	ns	ns	R^2^ adjusted = 0.226
						F ratio = 10.96
						*P* = 0.002
	T_MIN_	ns	ns	ns	ns	R^2^ adjusted = n/a
						F ratio = n/a
						*P* = n/a
	N_ER_	ns	0.11 (0.64) 0.529	ns	ns	R^2^ adjusted = −0.018
						F ratio = 0.40
						*P* = 0.529
	TB	ns	463.23 (2.56) 0.015	ns	ns	R^2^ adjusted = 0.141
						F ratio = 6.56
						*P* = 0.015

T_TTS_ – total test solving time; T_MIN_ – minimal time of particular test solving; N_ER_ – total number of wrong reactions; TB – total ballast time; BMI – body mass index; Hb – hemoglobin; hs-CRP – high sensitivity C-reactive protein; FC – faecal calprotectin; CDAI – Crohn’s disease activity index; ns – variables were not included in the model due to statistical insignificance (*P* > 0.1).

^a^B-value indicates regression coefficient of the independent variable.

^b^t-value indicates t-statistic value.

**Table 7:** Multiple linear regression analysis of selected variables as independent predictors of CRD parameters in the UC patients

Test type		BMI	Age	Duration of education	Mayo/DAI	Overall
		B^a^ (t^b^) *P*				
CRD 311	T_TTS_	ns	411.72 (6.44) <0.001	ns	ns	R^2^ adjusted = 0.304
						F ratio = 15.82
						*P* < 0.001
	T_MIN_	ns	3.89 (3.74) 0.002	ns	ns	R^2^ adjusted = 0.343
						F ratio = 18,79
						*P* < 0.001
	N_ER_	ns	ns	ns	ns	R^2^ adjusted = n/a
						F ratio = n/a
						*P* = n/a
	TB	ns	92.73 (2.23) 0.039	ns	ns	R^2^ adjusted = 0.077
						F ratio = 3.84
						*P* = 0.059
CRD 11	T_TTS_	ns	2885.00 (3.78) 0.001	ns	ns	R^2^ adjusted = 0.296
						F ratio = 8.16
						*P* = 0.001
	T_MIN_	-35.93 (-1.60) 0.130	35.74 (4.98) <0.001	-41.16 (-4.11) 0.68	ns	R^2^ adjusted = 0.575
						F ratio = 16.36
						*P* < 0.001
	N_ER_	ns	ns	ns	ns	R^2^ adjusted = n/a
						F ratio = n/a
						*P* = n/a
	TB	ns	1834.20 (2.85) 0.011	ns	ns	R^2^ adjusted = 0.087
						F ratio = 4.23
						*P* = 0.049
CRD 411	T_TTS_	ns	978.27 (4.54) <0.001	ns	ns	R^2^ adjusted = 0.226
						F ratio = 10.96
						*P* = 0.002
	T_MIN_	ns	9.51 (6.14) <0.001	ns	ns	R^2^ adjusted = n/a
						F ratio = n/a
						*P* = n/a
	N_ER_	ns	ns	ns	ns	R^2^ adjusted = -0.018
						F ratio = 0.40
						*P* = 0.529
	TB	ns	589.95 (3.34) 0.004	-197.13 (-4.51) 0.039	ns	R^2^ adjusted = 0.141
						F ratio = 6.56
						*P* = 0.015

T_TTS_ – total test solving time; T_MIN_ – minimal time of particular test solving; N_ER_ – total number of wrong reactions; TB – total ballast time; BMI – body mass index; Hb – hemoglobin; hs-CRP – high sensitivity C-reactive protein; FC – faecal calprotectin; Mayo/DAI – Mayo score/disease activity index for ulcerative colitis; ns – variables were not included in the model due to statistical insignificance (*P* > 0.1).

^a^B-value indicates regression coefficient of the independent variable.

^b^t-value indicates t-statistic value.

### The within and between-group differences in test solving times regarding different test phases of CRD tests

There was a significant difference between the IBD and control group regarding time responses in different test phases (P < 0.05). Moreover, there was a significant within-group difference in time responses between different test phases (P < 0.001) (Table 8).

**Table 8:** Comparison of test solving duration between different test phases of the CRD tests.

Test type	Repeated Measures 2-factor ANOVA		
	*Greenhouse-Geisser method*	*Between-group effects*	*Within-group effects*
CRD 311	*ε* = 0.527	F = 4.80	F = 63.69^a^
		*P* = 0.030	*P* < 0.001^a^
CRD 11	*ε* = 0.919	F = 7.71	F = 6.36
		*P* = 0.006	*P* < 0.001
CRD 411	*ε* = 0.880	F = 6.49	F = 62.99
		*P* = 0.012	*P* < 0.001

Grouping variable: IBD group vs. control group; Repeated measurement variables: T_1_; T_2_; T_3_; T_4_.

^a^Greenhouse-Geisser correction

T_1_ – time of reactions in the first test phase; T_2_ – time of reactions in the second test phase; T_3_ – time of reactions in the third test phase; T_4_ – time of reactions in the fourth test phase.

## Discussion

Our study has shown that patients with IBD have impaired neurocognitive and psychomotor function compared to the control subjects in all measured domains including perceptive abilities, convergent thinking and sophisticated operative thinking. The aforementioned findings were present irrespective of age, duration of education and BMI. To our knowledge, this is the first study that used the CRD-series for cognitive and psychomotor evaluation in IBD patients. Even though there are several studies evaluating cognitive function in patients with IBD, our study adds important findings to the existing evidence due to the important methodological strength of the CRD-series tests. To date, only Berrill *et al*. performed a similar, fully computer-based study on an entire population of IBD patients. However, they did not establish an independent significant difference in cognitive and psychomotor parameters between the observed group and healthy subjects reassuring the need for further studies^18^. Whether IBD *per se* leads to cognitive and psychomotor impairment was not definitely and consistently established.

Cognitive function has been the focus of numerous studies in different patient populations^8,13,32–34^ representing an important clinical outcome affecting all areas of life and disease^5^. However, validated studies on the IBD population are lacking. Moreover, cognitive heterogeneity aggravates measurement and result validation. Therefore, different performance tests have been used for the assessment of the subject’s cognitive function^13,15,18^.

Our study utilized CRD tests for detailed neurocognitive and psychomotor assessment. Multiple studies have shown that the CRD-series tests offer validated, reliable, objective and reproducible assessment of an individual’s cognitive and psychomotor characteristics^30–34^. Its language independence, versatility and integrated test generator minimise psychometric bias allowing for multiple retesting in different age and ethnic categories^30^. Most importantly, the high sensitivity of CRD tests allows the detection of minimal changes in cognitive and psychomotor performance.

Several questionnaire-based studies assessed cognitive function in IBD patients. Attree *et al*. conducted a study on a group of IBD patients with interesting results showing a specific deficit in verbal IQ among the studied group. Furthermore, they found deficits in the object recognition domain of cognitive functioning, with paradoxical superiority in the spatial recognition domain among IBD patients^12^. These findings were subsequently confirmed by Dancey *et al*.^15^. Similarly, Kennedy *et al*. did not show visuospatial memory impairment among CD patients Yet, they found a cognitive deficit in the Stroop test which partially evaluates the object recognition function^9^. Moreover, a study on adolescent IBD patients showed borderline significant findings of minor verbal deficits in comparison to age-matched patients with juvenile idiopathic arthritis. However, they did not find evidence of global cognitive impairment in adolescents with IBD^19^. Similarly, Wells *et al*. did not find any cognitive impairment in the IBD sample using the same Wechsler Adult Intelligence Scale as in the aforementioned studies^14^.

Moreover, Golan *et al*. and Langenberg *et al*. questioned cognitive and psychomotor function utilizing similar computerized tests on a sample of patients with CD. A study by Golan *et al*. found declines in verbal function and data processing speed compared to the normative data of healthy subjects^16^. Langenberg *et al*. provided insights that CD patients exhibit subtle cognitive impairment displayed by a prolonged response time in both the mental processing and decision making domain, but not with error rates^17^. Consistent findings for the aforementioned cognitive domains were obtained in our study indicating compensatory prolonged decision making and data processing time, with a preserved overall efficiency and accuracy in simple cognitive tasks.

Finally, a related computer-based study by Berrill *et al*. did not find any statistically significant difference in cognitive performance between IBD patients and control subjects after adjustment for covariates. Importantly, they did not measure sophisticated operative thinking and complex psychomotor coordination, which we assessed in our study^18^.

Generally, our study has shown impaired overall cognitive and psychomotor function in patients with IBD compared to healthy subjects, irrespective of age, duration of education and BMI. Specifically, our IBD patients exhibited longer T_TTS_ in all measured cognitive dimensions compared to healthy controls. In terms of mental chronometric principles, these results demonstrate the basic inferiority of the IBD group in neurocognitive and psychomotor performance. Explicitly, it indicates slower mental processing speed, decreased mental endurance and prolonged data analysis in the domains of perceptive abilities, convergent thinking, decision making and complex operative thinking. Consistent findings of prolonged response times in the domain of perceptive abilities and decision making speed were shown by Langenberg *et al*. on a sample of CD patients^17^. Finally, IBD patients showed decreased cognitive potential and mental capacity in the domains of perception and sophisticated operative thinking in comparison to the control group.

Different complexity of the CRD tests could provide an insight into the level of cognitive impairment. As reported, simple forms of cognitive tests (CRD-311 and CRD-11) revealed a stable performance (TB) and equal accuracy (N_ER_) in both study groups. The main deteriorations in the aforementioned tests among IBD patients were found in the mental processing speed, mental endurance and data analysis (T_TTS_; T_MIN_) domains. However, performance in the most complex test CRD-411, which requires operative thinking and harmonized eye-hand-leg coordination revealed significant additional impairment in answer accuracy, efficiency, attention, alertness (N_ER_), mental stability and cognitive fluctuation (TB) domains among IBD patients. Specifically, IBD patients exhibited equal error rates with prolonged response times in comparison to control subjects when analysing the results of simple CRD tests. However, the most complex CRD test revealed increased error rates, cognitive instability and prolonged response times as well among the IBD group. Preceding findings suggest compensation in simple cognitive tests on the account of prolonged mental processing, problem solving and decision making (T_TTS_). Therefore, in terms of accuracy and error rates (N_ER_), it can be stated that IBD patients may exhibit latent cognitive impairment in simple cognitive functions and manifested cognitive impairment regarding complex cognitive functions. Aforementioned outcomes could suggest that complex cognitive functions are more affected by the disease process. Similar findings were presented in a related study which found prolonged global cognitive response times, but unaltered error rates, indicating subtle cognitive impairment in patients with CD^17^.

Moreover, there was a significant difference between the IBD and control group regarding time responses in different test phases. The latter could indicate fragile cognitive stability, earlier cognitive fatigue and susceptibility to mental exhaustion among IBD patients. However, further studies are needed.

Finally, our study suggests that IBD *per se* deteriorates cognitive and psychomotor dysfunction, independent of ageing, education level and BMI status. The mechanism for cognitive and psychomotor dysfunction among IBD patients in our study can eventually be understood as a consequence of an intrinsic disease process. However, further studies are necessary for the establishment of a true causal relationship.

The pathophysiological basis for cognitive impairment in IBD patients has been investigated by Petruo *et al*.^35^. They have demonstrated selective functional alterations at the level of response selection in the fronto-striatal pathway. The aforementioned brain regions are especially important for cognitive flexibility, leading to a task-switching deficit in IBD patients. Interestingly, the neurophysiological analysis did not reveal functional deficits in brain regions important for working memory, the attentional selection process and perceptual categorization^35^. Conversely, our findings of aggravated CRD performance in the used tests indirectly point to a dysfunction in those cognitive components with the exception of memory.

Except for the functional neuropsychological deteriorations, scarcely available studies undoubtedly report morphologic pathologic changes in IBD patients as well. Hollerbach *et al*. assessed neurophysiological function and morphologic changes in IBD patients using evoked potentials and magnetic resonance imaging. The study had shown prolonged p300 wave latency and focal white matter lesions equivalent to short-term memory dysfunction and sensory perception disturbances. The aforementioned functional changes were mostly present in cortical postcentral regions corresponding to the somatosensory cortex, which is important for sensory data processing leading to perception deficit, which can correspond to the findings from our study^36^. These pathophysiological and morphologic changes are certainly interconnected, possibly by impaired neurogenesis and reduced neuroplasticity in IBD patients.

Whether disease activity plays a role in cognitive dysfunction is not definitely established. Data from our study did not reveal any significant correlation between CRD parameters and disease activity indices in any tested domain. Furthermore, we did not establish any significant correlation between FC and cognitive function. Consistent findings were reported by several studies^14,17,18^. However, Langenberg *et al*. established an interesting finding of an independent correlation between FC and better cognitive response times in the mental processing domain^17^. On the contrary, Golan *et al*. have shown a significant correlation of worse global cognitive function and CDAI^16^. Since the available studies offer indecisive results, future studies are needed.

Albeit it has been shown that systemic inflammatory parameters do not perfectly provide insight into the degree of intestinal inflammation, epidemiological studies indicate that systemic inflammation can deteriorate cognitive function^37,38^. However, no correlation was generally found between CRD variables and hs-CRP levels in our study. Similarly, a related study did not establish a significant correlation between CRP and global cognition^16^. On the contrary, Langenberg *et al*. found an independent positive correlation of hs-CRP with both mental processing and decision making time^17^. Nevertheless, levels of hs-CRP levels did not affect cognitive performance in our study.

Moreover, Hb concentrations were generally not correlated with CRD parameters in our study. Similarly, Wells *et al*. did not find any significant correlation of Hb concentrations and cognitive function. Moreover, they did not observe improvements in cognitive function with the treatment of anemia in IBD patients^14^. However, Golan *et al*. found a significant correlation between Hb levels and better cognitive performance^16^. On the contrary, a related study on a CD sample has shown an independent correlation of plasma Hb levels with slower mental processing speed^17^. Nonetheless, there is no evidence of the influence of Hb levels on cognitive function in our study.

Moreover, it was previously shown that ageing deteriorates cognitive and psychomotor function^39^. Consistently, we have established a significant correlation of age with worse cognitive performance in most CRD parameters. However, a related study did not establish any significant correlation between age and cognition^17^. Moreover, findings by Attree *et al*. and Dancey *et al*. were adjusted for age showing that cognitive verbal deficit is independent of age^13,15^. Importantly, similar to previous studies, our sample did not include geriatric subjects so the aforementioned findings cannot be applied to elder IBD patients^13,15,17^. However, there were no differences in age and gender between the observed groups in our study, so we can state that groups were adequately age- and sex-matched. Finally, it should be noted that age was included as a covariate in the ANCOVA model in our study.

Whether there is a discrepancy in cognitive performance between CD and UC patients is not well established, but the available evidence does not support the conjecture of disparity. Likewise, we have not established a significant difference in cognitive performance among patients with CD and UC, which is consistent with related studies^13,18^.

All of the above findings emphasize the cognitive burden of IBD on daily executive functions such as working abilities, especially in the fields, which require stable performance, strong concentration and repetitive operations. It is necessary to underline that, depending on the complexity, the used CRD tests correspond and simulate usual daily functions – shopping abilities, job tasks, vehicle driving, etc.^32^. Since even subtle impairment in reaction time increases the risk of traffic road and workplace accidents, these findings in the IBD population are of definite clinical significance. However, these assumptions could not be confirmed since appropriate studies are lacking. Nonetheless, self-awareness in these patients could eventually encourage job prequalifications and avoidance of prolonged drive, eventually leading to accident prevention. Additionally, prevention and early treatment could theoretically intercept cognitive deterioration in these patients. Finally, cognitive dysfunction could lead to problems with therapeutic compliance and dietary discipline, and therefore to circulus vitiosus of the IBD and cognitive impairment.

Similar to other studies, our study has several limitations. Firstly, our research design was cross-sectional, so it was not possible to establish any causal relationship or follow longitudinal cognitive alterations. Further, our sample mostly involved patients with IBD of low to moderate activity so we are not able to extrapolate our findings on the entire IBD population, yet we can only speculate. Further, the CRD-series are not specifically designed for the IBD population, but are rather generic instruments for cognitive and psychomotor function assessment. Moreover, our study did not assess verbal function and memory, so we were not able to fully compare these cognitive domains with the few available studies. Additionally, the impact of mood disorders such as depression canot be fully excluded since we haven’t used any depression quantification score. Moreover, we cannot fully exclude the influence of anxiety and fatigue on the cognitive performance in our study. This should be additionally evaluated in further studies on an IBD population with CRD utilization. Still, all subjects denied the presence of mood disorder in a self-reported questionnaire and none of them had a previous clinical diagnosis or clinically evident signs of depression, anxiety and chronic fatigue. Finally, normative data for CRD tests are not established, so it is impossible to validate control group results.

Since this is the first study using the CRD instrument on an IBD population, it is difficult to compare our results with other studies. However, the categorization of study results in different cognitive domains alleviates the interpretation and allows for more direct comparison. There is a substantial need for an objective, non-invasive and standardized neuropsychological test battery in the IBD population^18^. With respect to the aforementioned methodological advantages of the CRD tests, it may be desirable to encourage further comprehensive CRD studies on IBD patients^30–34^.

In conclusion, our study has shown impaired neurocognitive and psychomotor function in patients with IBD compared to control healthy subjects, especially in mental processing speed and mental endurance of perceptive abilities, convergent thinking and sophisticated operative thinking, which was independent of age, duration of education and BMI. Further research is necessary to clarify the significance of these findings.

## Data Availability

We disclose any restrictions on the availability of data, materials and associated protocols.
